# Supplementary material for: Conditional targeting of phosphatidylserine decarboxylase to lipid droplets
Source: Biol Open. 2021 Mar 3;10(3):bio058516. doi: 10.1242/bio.058516 (PMC7938800; doi:10.1242/bio.058516)
Supplement: Supplementary information [file biolopen-10-058516-s1.pdf]

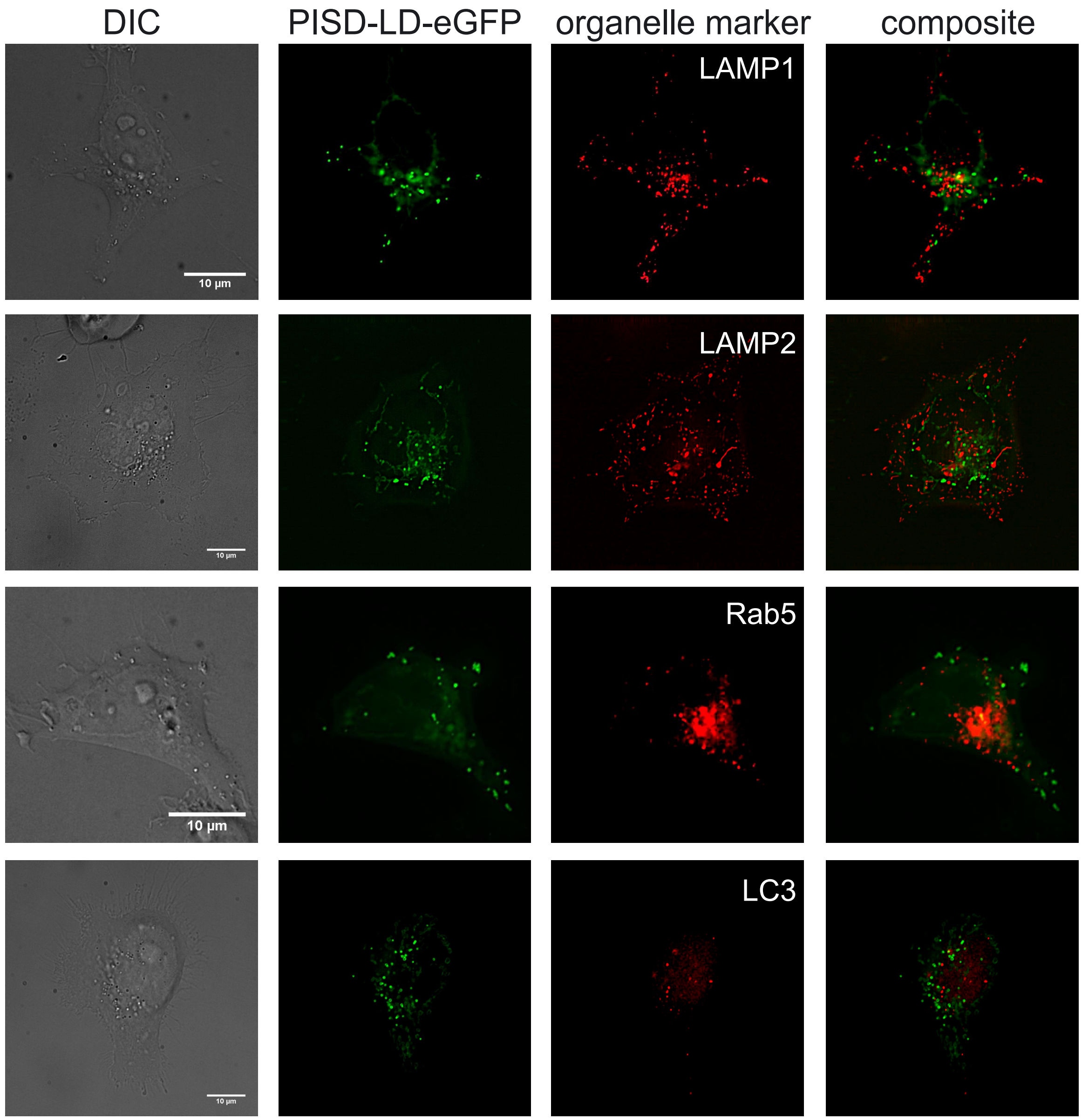


**Figure S1:** PISD-LD-GFP does not localize to organelles of the endo-lysosomal system of the autophagy protein, LC3. Hela cells were co-transfected with 50ng pPISD-LD-eGFP vector and plasmids encoding RFP-tagged forms of the indicated lysosomal proteins (500ng pLAMP1-RFP or 500ng pLAMP2-RFP), an early endosomal protein, RFP-Rab5 (500ng pRab5-RFP) or a marker of the autophagosome, RFP-LC3 (600ng of pLC3-RFP). Cells were visualized by deconvolution fluorescence microscopy beginning 16 hours after transfection. Images shown are a single focal plane from a Z series. The scale bars represent 10 µm.

Biology Open • Supplementary information
